# Barriers and Associated Factors to Writing Case Reports Among Japanese General Medicine Physicians: A Cross‐Sectional Study of the Japan Primary Care Association Members

**DOI:** 10.1002/jgf2.70138

**Published:** 2026-06-01

**Authors:** Tsuneaki Kenzaka, Shinsuke Yahata, Naoya Mizutani, Ayako Kumabe, Ryo Fujiwara, Moe Kyotani, Koki Kosami, Hiroyuki Mori

**Affiliations:** ^1^ Division of Community Medicine and Career Development Kobe University Graduate School of Medicine Kobe Japan; ^2^ Department of Internal Medicine Hyogo Prefectural Tamba Medical Center Tamba Japan; ^3^ Department of General Internal Medicine Hyogo Prefectural Harima‐Himeji General Medical Center Himeji Japan; ^4^ Department of General Medicine Toyooka Public Hospital Toyooka Japan; ^5^ Division of Public Health, Center for Community Medicine Jichi Medical University Shimotsuke Japan

**Keywords:** barriers, clinical message, general practitioner, generalist, Japan Primary Care Association, writing case reports

## Abstract

**Background:**

Case reports are an important academic output for general medicine physicians; however, publication rates in Japan remain low, and barriers to preparing case reports have not been fully clarified. Thus, we aimed to identify barriers perceived by Japanese general medicine physicians when writing case reports.

**Methods:**

A web‐based questionnaire survey was administered to physician members of JPCA between November and December 2021. Sixteen potential barriers identified in previous studies were assessed using an 11‐point Likert scale (0–10). Participants were categorized into two groups based on their experience writing case reports. Group comparisons were performed using the Mann–Whitney test and chi‐squared test. Logistic regression analyses, adjusted for age and sex, were conducted to estimate adjusted odds ratios (aORs) for each barrier.

**Results:**

Overall, 315 physicians responded to the survey (response rate: 10.2%). Of these, 159 (50.4%) had experience in writing case reports. Fifteen of the 16 barrier items scored significantly higher among inexperienced physicians. The aORs were significantly elevated for 13 items, excluding financial factors. The major barriers included lack of sufficient medical documentation to write a report (aOR: 7.99), unfamiliarity with writing case reports (aOR: 6.44), not recognizing suitable cases (aOR: 4.79), and difficulty identifying the main case points and clinical messages (aOR: 3.80). Experienced physicians not “having adequate time to write” remained the main obstacle (69.8%).

**Conclusions:**

Barriers to writing case reports vary markedly by experience. Systematic education, strengthened mentorship, and organizational support for protected academic time are essential to promoting case report writing among general medicine physicians.

## 1 Introduction

Case reports are widely recognized as valuable educational resources that foster clinical reasoning, reflective practice, and lifelong learning among general medicine physicians [1, 2, 3, 4]. They also play an important function in documenting rare medical conditions and generating new clinical insights [5, 6]. Despite these advantages, scholarly output among primary care physicians remains limited in several countries, including Japan. For instance, only 3.8% of abstracts presented at the Japan Primary Care Association (JPCA) Annual Meeting were subsequently published, with a small proportion comprising case reports [7, 8]. This suggests that important clinical knowledge acquired through daily practice is not being adequately disseminated.

International studies have consistently identified barriers to scholarly activity among primary care physicians, including insufficient time, lack of mentorship, limited research training, and inadequate institutional support [9, 10, 11, 12]. Nevertheless, most of these studies focus on general research engagement rather than the specific task of case report writing. Authoring case reports requires distinct competencies—including selecting appropriate cases, articulating key clinical messages, and preparing comprehensive medical documentation—that differ from those required for original research articles [1, 13]. Therefore, barriers to case report writing cannot be fully inferred from studies examining broader academic activities.

In Japan, few exploratory studies have investigated barriers to writing case reports, with most studies involving small samples and physicians from diverse specialties [14]. Consequently, the particular obstacles encountered by Japanese general medicine physicians—who are expected to participate in case‐based learning and contribute to clinical knowledge—remain insufficiently understood. Moreover, the extent to which these barriers differ between physicians with and without prior experience in case report authorship is unclear, despite the likelihood that experience influences both perceived difficulty and support requirements.

To address these knowledge gaps, we conducted a nationwide survey of JPCA physician members to systematically identify perceived barriers to case reporting and examine how these challenges vary by prior writing experience.

## 2 Materials and Methods

### 2.1 Study Design

This cross‐sectional study was approved by the Ethics Committee of the Hyogo Prefectural Tamba Medical Center (approval number: Tan‐I 1304).

### 2.2 Participants

All physician members of the JPCA were eligible to participate.

### 2.3 Survey Methodology

A web‐based questionnaire was distributed via the JPCA mailing list between November and December 2021. The questionnaire collected data on sex, age, experience in writing case reports, and (if applicable) the number of case reports written, participation in case report workshops, presence of a research mentor, availability of literature search resources, institutional financial support for academic writing, external research funding, and membership in research‐support social media groups.

We assessed 16 potential barriers to case report writing using an 11‐point Likert scale (0 = “not a barrier at all,” 5 = “neither a barrier nor a facilitator,” and 10 = “very much a barrier”). These barrier items were initially identified through our research group's prior exploratory studies, which were presented at academic meetings before 2021 and subsequently published in an expanded format [14]. Scores ranging from 0 to 5 were categorized as “no barrier,” whereas scores of 6–10 were designated as “barrier,” indicating a shift away from neutrality toward perceiving the item as a meaningful obstacle. The Barrier items included the following:

#### 2.3.1 Factors Related to Paper Writing

- Identify cases suitable for publication as case reports.
- Sufficient medical documentation to compose a report.
- Understand the structure and requirements for writing a case report.
- Determine the key case points and clinical messages.
- Receive guidance or support from mentors.

#### 2.3.2 Factors Related to Literature and Cost

- Understand how to conduct a literature search
- Understand how to obtain the literature
- Financial cost of accessing literature.
- Cost of proofreading
- Cost of publication

#### 2.3.3 Factors Related to Time, Motivation, and Other Issues

- Insufficient time available for writing
- Low motivation to write.
- Difficulties with English language proficiency.
- Trouble selecting a suitable journal for submission.
- Uncertainty about the need for ethical review.
- Lack of knowledge on how to apply for an ethical review.

The full Japanese version of the questionnaire is provided in Supplementary File S1.

### 2.4 Development of the Survey Instrument

The 16 barrier items included in the questionnaire were originally derived from our previous exploratory study [14], which revealed common challenges physicians encounter when writing case reports. These items were refined through discussions among general medicine educators experienced in supervising case report writing. While no formal theoretical framework exists for barriers to case report writing, established concepts in academic writing—such as selecting cases, identifying messages, ensuring quality documentation, and accessing mentorship—informed the item selection.

Prior to distribution, the questionnaire was pilot tested with five general medicine physicians to evaluate its clarity, relevance, and comprehensiveness. Minor wording changes were made based on their feedback to enhance face validity and ensure each item reflected a distinct aspect of the case report writing process. Although certain items may appear conceptually related—such as “knowing how to write a case report,” “determining the main message,” and “recognizing suitable cases”—they were intentionally treated as independent, as these tasks represent distinct steps in the workflow. Identifying a publishable case, extracting its clinical message, and applying appropriate writing techniques require different cognitive processes and skill sets; our previous qualitative data suggested that beginners often struggle with these steps individually.

### 2.5 Data Analysis

Descriptive statistics were calculated. Participants were divided into two groups based on their experience with case report writing. Group comparisons were performed using the chi‐squared test for categorical variables and the Mann–Whitney test for Likert‐scale barrier scores, as these data were not normally distributed (*p* < 0.05 indicated statistical significance). These statistical tests were applied exclusively to the barrier items (Table 2). The descriptive statistics are presented in Table 1. Scores of 0–5 were classified as “no barrier,” whereas scores of 6–10 were classified as “barrier”.

Logistic regression analyses adjusted for age and sex were conducted to estimate the crude odds ratios (ORs) and adjusted odds ratios (aORs) for each barrier. Additionally, sensitivity analyses were conducted using alternative cutoff values (≥ 5 and ≥ 7) to assess whether thresholds influence the results. Given that several potentially important confounders—such as academic activity during training or experience presenting at conferences—were not measured, the regression analyses were conducted to supplement, rather than substitute for, the comprehensive descriptive statistics. All statistical analyses were performed using IBM SPSS Statistics version 29.0 (IBM Corp., Armonk, NY, USA).

## 3 Results

At the start of the survey, 3085 physicians were registered on the mailing list, and 315 responded to the web questionnaire (response rate: 10.2%). Of these, 239 (75.9%) were male, and the median age was 42 years (interquartile range: 36–52 years). Case report writing experience was reported by 159 physicians (50.4%), with a median of three written case reports (range: 1–80).

### 3.1 Baseline Characteristics

**FIGURE 1:**
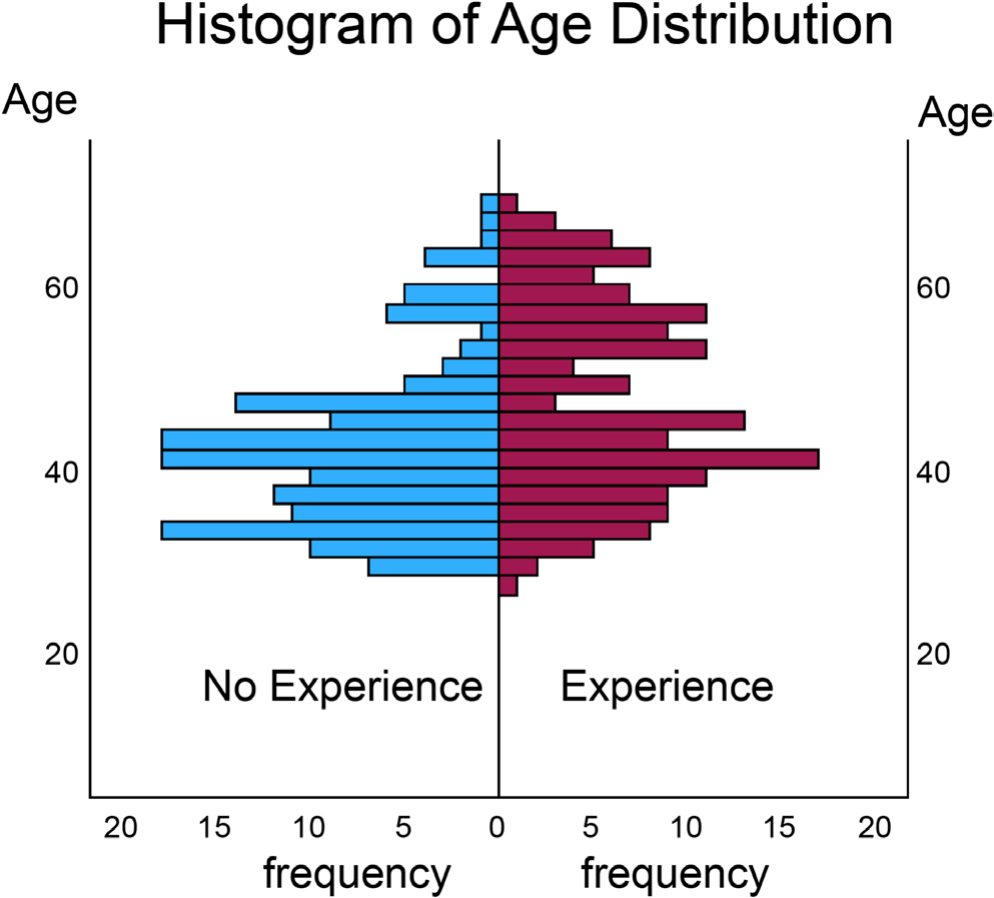
Histogram of age distribution stratified by case report writing experience. Age distribution of participants in 10‐year intervals, separated into physicians with and without case report writing experience. The overall distribution pattern is presented, highlighting differences in age structure between the groups.

At baseline, experienced physicians tended to be older and were more likely to have research mentors and institutional financial support (Table 1). Other characteristics showed similar distributions between the two groups (Figure 1).

**TABLE 1:** Comparison of basic characteristics and research support environments based on experience in writing case reports.

Item regarding potential barriers	No experience (*n* = 156)	Experience (*n* = 159)
Age	41 [34 to 46]	45 [38.5 to 56.5]
Male	107 (68.6%)	132 (83.0%)
Female	49 (31.4%)	27 (17.0%)
Participation in case report workshops	20 (12.8%)	25 (15.7%)
Presence of a research mentor	50 (32.1%)	69 (43.4%)
Availability of literature search resources	80 (51.3%)	96 (60.4%)
Institutional financial support	41 (26.3%)	60 (37.7%)
External research funding	19 (12.2%)	58 (36.5%)
Membership in research‐support social media groups	20 (12.8%)	16 (10.1%)

*Note:* Age is reported as the median and interquartile range.

### 3.2 Barrier Scores and Perceived Barrier Rates

Analyses of the median scores and perceived barrier rates across 16 items revealed that inexperienced physicians reported higher barrier scores across nearly all items (Table 2). In all comparisons, results are presented for inexperienced physicians followed by experienced physicians. The largest differences were observed in the early stages of the writing process. For example, “having sufficient medical documentation to write a report” differed markedly between groups (7 [5–8] vs. 3 [1.5–5]; barrier prevalence 72.4% vs. 23.9%). Similarly, “knowing how to write a case report” exhibited a substantial difference (8 [6–9] vs. 4 [2–6]; barrier prevalence 76.3% vs. 30.8%).

**TABLE 2:** Scores and proportions of perceived barriers to writing case reports (16 items).

Item regarding potential barriers	No experience (*n* = 156) median (Interquartile range)	Experience (*n* = 159) median (Interquartile range)	*p*	No experience: barrier *n* (%)	Experience: barrier *n* (%)	*p*
Recognizing cases suitable for a case report	7 [5 to 8]	3 [2 to 6]	< 0.001	108 (69.2%)	48 (30.2%)	< 0.001
Having sufficient medical documentation to write a report	7 [5 to 8]	3 [1.5 to 5]	< 0.001	113 (72.4%)	38 (23.9%)	< 0.001
Knowing how to write a case report	8 [6 to 9]	4 [2 to 6]	< 0.001	119 (76.3%)	49 (30.8%)	< 0.001
Determining the main case points and clinical message	8 [5 to 9]	5 [2 to 7]	< 0.001	115 (73.7%)	61 (38.4%)	< 0.001
Lacking a mentor or supporter	8 [5 to 9]	6 [3 to 8]	< 0.001	112 (71.8%)	86 (54.1%)	0.001
Knowing how to search the literature	6 [3 to 8]	4 [1 to 5.5]	< 0.001	83 (53.2%)	40 (25.2%)	< 0.001
Knowing how to obtain literature	6 [3 to 8]	4 [2 to 7]	< 0.001	83 (53.2%)	53 (33.3%)	< 0.001
Financial cost of accessing literature	5 [2 to 7]	5 [1 to 7]	0.019	73 (46.8%)	61 (38.4%)	0.130
Cost of proofreading	6 [4 to 8]	5 [3 to 7]	0.036	86 (55.1%)	78 (49.1%)	0.281
Cost of publication	5 [4 to 7.25]	5 [3 to 7]	0.278	77 (49.4%)	75 (47.2%)	0.697
Having adequate time to write	8 [7 to 10]	7 [5 to 9]	< 0.001	133 (85.3%)	111 (69.8%)	0.001
Lacking motivation to write	8 [6 to 9]	5 [3 to 8]	< 0.001	118 (75.6%)	79 (49.7%)	< 0.001
Difficulty with English	8 [5 to 9]	6 [2 to 8]	< 0.001	108 (69.2%)	80 (50.3%)	< 0.001
Selecting an appropriate journal for submission	8 [5 to 9]	6 [3 to 8]	< 0.001	116 (74.4%)	88 (55.3%)	< 0.001
Determining whether ethical review is required	7 [5 to 9]	5 [2 to 7.5]	< 0.001	99 (63.5%)	71 (44.7%)	< 0.001
Knowing how to apply for ethical review	7 [4.75 to 8]	5 [2 to 8]	< 0.001	101 (64.7%)	70 (44.0%)	< 0.001

*Note:* Comparison between physicians with and without case report writing experience.

Other major differences included “recognizing suitable cases” (7 [5–8] vs. 3 [2–6]; barrier prevalence 69.2% vs. 30.2%), “determining the main clinical message” (8 [5–9] vs. 5 [2–7]; barrier prevalence 73.7% vs. 38.4%), and “lacking mentorship” (8 [5–9] vs. 6 [3–8]; barrier prevalence 71.8% vs. 54.1%).

Among experienced physicians, “having adequate time to write” remained the most prominent barrier (7 [5–9]; barrier prevalence 69.8%), indicating that time constraints persist regardless of experience (Figures S1–S16).

### 3.3 Logistic Regression Analysis

Logistic regression analyses further highlighted differences between the groups (Table 3). The strongest associations with inexperience were observed for the following factors:

- Having sufficient medical documentation to write a report (aOR = 7.99; 95% confidence interval [CI]: 4.73–13.50)
- Knowing how to write a case report (aOR = 6.44; 95% CI: 3.86–10.75)
- Recognizing cases suitable for a case report (aOR = 4.79; 95% CI: 2.91–7.89)
- Determining the main case points and clinical messages (aOR = 3.80; 95% CI: 2.32–6.23)

**TABLE 3:** Crude and adjusted odds ratios for barriers to writing case reports.

Item regarding potential barriers	Crude OR (95% CI)	*p*	Adjusted OR (95% CI)	*p*
Recognizing cases suitable for a case report	5.20 (3.22–8.41)	< 0.001	4.79 (2.91–7.89)	< 0.001
Having sufficient medical documentation to write a report	8.37 (5.04–13.88)	< 0.001	7.99 (4.73–13.50)	< 0.001
Knowing how to write a case report	7.22 (4.38–11.90)	< 0.001	6.44 (3.86–10.75)	< 0.001
Determining the main case points and clinical message	4.51 (2.79–7.27)	< 0.001	3.80 (2.32–6.23)	< 0.001
Lacking a mentor or supporter	2.16 (1.35–3.45)	0.001	1.96 (1.21–3.20)	0.007
Knowing how to search the literature	3.38 (2.10–5.45)	< 0.001	3.13 (1.90–5.16)	< 0.001
Knowing how to obtain literature	2.27 (1.44–3.59)	< 0.001	2.15 (1.34–3.46)	< 0.001
Financial cost of accessing literature	1.41 (0.92–2.21)	0.131	1.28 (0.80–2.05)	0.301
Cost of proofreading	1.28 (0.82–1.99)	0.281	1.19 (0.75–1.88)	0.473
Cost of publication	1.09 (0.70–1.70)	0.697	1.04 (0.66–1.66)	0.857
Having adequate time to write	2.50 (1.43–4.37)	0.001	2.31 (1.30–4.14)	0.005
Lacking motivation to write	3.15 (1.95–5.08)	< 0.001	3.23 (1.95–5.34)	< 0.001
Difficulty with English	2.22 (1.40–3.52)	< 0.001	2.24 (1.38–3.62)	< 0.001
Selecting an appropriate journal for submission	2.34 (1.45–3.77)	< 0.001	2.01 (1.22–3.30)	< 0.001
Determining whether ethical review is required	2.15 (1.37–3.38)	< 0.001	2.08 (1.30–3.34)	< 0.001
Knowing how to apply for ethical review	2.34 (1.48–3.68)	< 0.001	2.38 (1.48–3.84)	< 0.001

*Note:* Logistic regression adjusted for age and sex (Barrier = Score: 6–10).

Abbreviations: 95% CI, 95% confidence interval; OR, odds ratios.

These results demonstrate that inexperienced physicians face their greatest challenges in acquiring the foundational skills required for effective case report writing. In comparison, financial considerations did not differ significantly between the groups.

Sensitivity analyses were performed using alternative cutoff values (≥ 5 and ≥ 7) alongside the primary threshold of ≥ 6. While aORs varied with threshold, the overall trends in OR magnitude and the statistical significance associated with each barrier remained consistent, thus supporting the robustness of the study's outcomes. Detailed results of the sensitivity analyses are presented in Table S1.

## 4 Discussion

This study is the first nationwide survey in Japan to examine the barriers to writing case reports among Japanese general medicine physicians. However, the absolute number of respondents remains modest relative to the total general medicine physician population in Japan. While our previous research [14] was limited to an exploratory analysis of a small subset of participants, this study targeted a broader population of JPCA members, enabling an examination of the current state of academic activities in general practice.

The key findings of this study demonstrate that the barriers physicians face when writing case reports vary markedly with their prior experience. Inexperienced physicians reported significantly higher barriers across most items, particularly those related to the early stages of the writing process, such as recognizing cases suitable for a case report, having sufficient medical documentation, knowing how to write a case report, formulating the clinical message, and finding mentorship. Research worldwide highlights that structured mentorship is a key determinant of scholarly productivity among primary care physicians and early‐career clinicians. Evidence from several countries confirms that formal mentorship programs encourage research engagement, increase publication output, and lessen perceived barriers to academic writing. Our study's observation of differences in mentorship availability aligns with these global trends. Inexperienced doctors lacking mentorship may struggle more with selecting cases, developing messages, and preparing manuscripts, underscoring the need for structured and accessible mentorship within general medicine training. This supports our earlier research [14], which showed that beginners find “which case to select” and “what message to present” challenging and is confirmed by a larger dataset. Inexperienced physicians may perceive “having sufficient medical documentation” as a major barrier, likely due to uncertainty about the details required for publication. If critical information—such as diagnostic rationale, differential diagnoses, treatment decisions, or prognostic considerations—is not fully documented, authors may feel unable to defend their clinical decisions or address reviewers' questions. This belief arises from gaps in documentation practices, limited knowledge of journal expectations, or misconceptions about what constitutes acceptable case report material.

Experienced physicians reported fewer barriers, potentially due to factors like earlier exposure to mentorship, self‐directed learning opportunities, or existing skills in case selection and writing. However, since this study used a cross‐sectional design, it is unclear whether experience reduced these barriers or whether those already perceiving fewer barriers were simply more likely to participate in case report writing. Notably, “having adequate time to write” remained a significant barrier for experienced physicians (69.8%). This emphasizes how clinical duties often restrict academic activities, making individual‐level solutions insufficient.

From an organizational leadership perspective, several practical strategies may help strengthen case report writing. These include reserving protected academic time each week, forming internal writing teams or mentor pairing, simplifying administrative procedures related to ethics review, and providing centralized access to literature search resources or editorial support. These simple structural adjustments may enable healthcare institutions to balance clinical demands with scholarly development.

When interpreting these findings, structural and organizational influences warrant attention. International research on primary care consistently shows that heavy workloads, a lack of protected academic time, and institutional culture substantially limit clinicians' engagement in scholarly activities. As reported by a UK survey, major barriers include a lack of capacity for additional tasks and concerns about the effort required; over 40% of staff lacked prior research experience, underscoring the difficulty of integrating academic tasks into routine practice [9]. Similarly, an Israeli national study found that heavy workload, administrative burdens, and the absence of dedicated time impede research, whereas protected time, administrative support, and training facilitate engagement [15].

Organizational culture also shapes participation: commentaries in family medicine emphasize the need to embed a “culture of curiosity” into routine clinical practice to normalize research [16]. Canadian qualitative studies highlight that supportive organizational structures—such as collaborative environments and equitable research pipelines—are essential for building sustainable research capacity [17]. Portuguese qualitative research also identifies structural barriers such as limited time, infrastructure, administrative systems, and ethics review processes, illustrating how institutions influence individual research behavior [18]. Cumulatively, these findings of inadequate time reflect broader systemic issues. Addressing these obstacles requires coordinated action at both institutional and policy levels, rather than relying solely on individual motivation or skill‐building.

Barriers related to ethical review processes merit further consideration. In Japan, requirements for the ethical review of case reports vary by institution, and local policies often lack clarity or consistency. Some institutions mandate formal review for any case publication, while others exempt single‐case reports or permit expedited reviews. This variability may create uncertainty among clinicians, especially those with less experience or limited familiarity with institutional procedures, and ambiguity about when ethical approval is necessary. Moreover, institutional review boards may differ in their interpretation of national guidelines, resulting in inconsistent expectations concerning documentation, consent, and review timelines. Such discrepancies likely contribute to the relatively high ORs observed for “determining whether ethical review is required” and “knowing how to apply for ethical review” in the current study. Establishing clear local policies, standardizing procedures, and providing accessible guidance could mitigate these perceived barriers.

Financial factors—including the costs of accessing literature, proofreading, and publication—did not differ significantly between groups. This suggests that the financial challenges are common regardless of experience level. However, inexperienced physicians may underestimate financial barriers due to limited exposure to the submission process and thus lack a practical understanding of potential expenses (e.g., literature access, proofreading, publication fees). While some inexperienced physicians may theoretically perceive financial costs as a barrier, many do not fully appreciate these expenditures in practice, which may reduce observable group differences despite the clear disparity in institutional academic funding (Table 1). Given that financial barriers were perceived similarly across experience levels, institution‐level or society‐level support may be particularly important. Potential strategies include subsidizing proofreading or publication fees, negotiating discounted rates with journals or editorial services, expanding institutional access to literature databases, and offering shared editorial assistance within departments. Even modest financial supports—such as covering initial submission fees or providing vouchers for language editing—can lower entry barriers for first‐time authors and encourage sustained scholarly activity.

Sensitivity analyses employing alternative cutoff values (≥ 5 and ≥ 7) were conducted to evaluate the robustness of the findings. Although aORs varied with different thresholds, the relative associations and the significance of each barrier remained consistent. These results confirm that the associations identified in this study are stable across variations in cutoff selection.

The results suggest that the focus of support for case report writing varies according to the writer's experience. For novice authors, comprehensive assistance with their initial case report is particularly critical, encompassing guidance on evaluating the case's significance, defining key points, instruction in writing methodology, and establishing mentorship frameworks. Targeted approaches informed by barriers identified in this study may be particularly beneficial for beginners. First, structured measures to enhance medical documentation are essential, as inadequate documentation was most strongly linked to inexperience. Providing templates for clinical reasoning, differential diagnosis, and key decision points can facilitate consistent capture of essential information during routine patient care. Second, educational interventions should prioritize case selection and message extraction to reflect significant disparities across groups. Interaction workshops using real cases, peer discussion formats, or supervised case selection sessions may foster skill development in these areas. Third, clear guidance regarding ethical review processes—including simplified flowcharts or standardized institutional policies—may mitigate uncertainty among first‐time authors. Furthermore, improving access to literature search resources and offering introductory training in literature retrieval may address considerable differences in literature‐related barriers.

Several educational interventions, including mentorship, have demonstrated efficacy in advancing case report writing skills. International literature underscores the value of structured writing workshops, standardized templates, peer‐support groups offering iterative feedback, and editorial assistance programs dedicated to manuscript refinement. These interventions not only enhance technical writing proficiency but also alleviate psychological obstacles through clear frameworks and collaborative engagement. Incorporating such educational initiatives into general medicine training programs may empower inexperienced physicians to overcome early‐stage barriers and complete their first case report. This aligns with elements emphasized in case report writing tips [13], such as “clarifying novelty” and “extracting the clinical message.”

Furthermore, case reports contribute significantly to the accumulation of knowledge regarding rare diseases and serve as foundational tools for clinical reasoning education [1, 2, 3, 4, 5, 6]. To broaden academic participation, implementing educational interventions that enhance motivation for writing case reports and establish robust support systems within academic societies and training institutions is warranted. Notably, an investigation into the time management strategies employed by physicians who have published numerous papers represents a promising avenue for future research.

## 5 Limitations

This study has several notable limitations. First, the 10.2% response rate introduces the possibility of response bias. Participating physicians may have exhibited a greater interest in academic activities or higher motivation to enhance scholarly skills than non‐respondents; conversely, those experiencing substantial barriers or limited academic engagement may have been less inclined to respond. Consequently, the perspectives captured in this study may not fully represent the JPCA membership. Previous questionnaire‐based surveys conducted in Japan among physicians without financial incentives have generally reported response rates of approximately 15% [19, 20, 21]. In contrast, web‐based surveys targeting members of the JPCA have demonstrated response rates ranging from 6% to 23% [22, 23]. Although our response rate falls within this range, it remains low for survey research, as rates exceeding 60% are generally preferable [22]. Therefore, the low response rate constitutes an important limitation, underscoring the need for future studies to enhance participation through refined recruitment strategies, repeated reminders, or multimodal survey approaches [22].

Second, the cross‐sectional design of the study precludes causal inferences. It is unclear whether accumulated experience mitigates perceived barriers or if physicians initially facing fewer barriers subsequently gained experience. Due to this study design, causal relationships between perceived barriers and case report writing experience cannot be established; observed associations should be interpreted as correlations rather than directional effects. Furthermore, reverse causality cannot be excluded. While findings indicate that experienced physicians were more likely to have research mentors and institutional support, such resources may have been acquired after initiating case report writing, rather than serving as original facilitators. This bidirectional relationship warrants careful consideration in interpreting the associations identified in this study.

Third, barriers were self‐reported and may have been influenced by recall or social desirability biases. Specifically, items related to financial considerations and ethical reviews may be challenging for physicians without actual submission experience to visualize concretely, potentially leading to their underestimation as barriers. Participants may unintentionally overestimate or underestimate certain obstacles due to memory, perception, or an inclination to present themselves in a favorable light. These biases could compromise the accuracy of reported barriers and should be considered when interpreting the findings.

Fourth, the study did not evaluate the quality or context of the case report writing experience. While the number of case reports was surveyed, information on academic maturity—such as guidance received, journal submissions, acceptance rates, or peer review involvement—was missing. These factors may influence perceptions of barriers; thus, future research should incorporate more comprehensive indicators of experience. Furthermore, prior exposure to academic writing during residency or postgraduate training was not assessed, which may substantially impact confidence and perceived barriers, functioning as a potential confounder. Physicians who participated in structured writing instruction or academic activities during training may perceive fewer barriers irrespective of subsequent case report writing experience. The lack of such information constrains our ability to fully account for educational background as a contributing factor.

Fifth, while adjusting only for sex and age when calculating the aORs, we may not have sufficiently controlled for other potential confounding factors that could influence barrier perception, such as work patterns, facility size, academic culture, workload, and educational systems. Differences in work environments could significantly impact perceptions of “having adequate time to write.” Additionally, relevant confounders, such as experience presenting at conferences, academic culture, or prior scholarly engagement, were not measured. Descriptive distributions depicted in histograms may offer a more direct illustration of underlying data. Defining scores ≥ 6 as indicating the presence of a barrier, based on the scale's neutral midpoint, may reduce information detail due to dichotomization. Nonetheless, sensitivity analyses applying alternative thresholds (≥ 5 and ≥ 7) yielded similar association patterns, underscoring robustness. Accordingly, logistic regression findings should be interpreted as exploratory and supportive rather than inferential, with primary emphasis placed on the descriptive presentation of raw data. Moreover, this study did not assess whether participants had prior experience presenting case reports at academic conferences—a factor likely to influence confidence, familiarity with writing processes, and perceived barriers.

In addition to these methodological considerations, temporal factors must be acknowledged. A significant interval has elapsed between the 2021 survey administration and the current submission, during which the research and educational environment has evolved substantially. The rapid advent of generative AI tools has transformed literature searches, manuscript drafting, and writing support for physicians. While these developments do not diminish the relevance of this study's findings, the identified barriers may differ from those encountered in the current environment. Future studies should reassess these barriers in light of evolving technologies and academic support systems.

Finally, this study focused on Japanese general medicine physicians, limiting its generalizability to physicians in other countries or specialties. Cultural backgrounds, educational frameworks, and academic support systems for case report writing vary markedly across countries and specialties, highlighting the need for future international comparative studies.

## 6 Conclusions

This study clarifies the main obstacles Japanese general medicine physicians face when writing case reports. Inexperienced physicians often struggle with issues related to the writing process, including recognizing suitable cases, collecting sufficient medical documentation, understanding how to write a case report, identifying main points and clinical messages, and attaining mentorship. Both a lack of time and a lack of motivation are significant challenges. Notably, even among experienced physicians, having adequate time to write remains the greatest barrier, indicating a structural challenge that is difficult to resolve solely through accumulated experience.

These results suggest that effective support for case report writing should include structured education and stronger mentorship for beginners, practical support for defining key points and writing methods, and organizational support that enables time allocation for academic activities. Promoting academic engagement in general practice requires ongoing efforts to highlight the value of case report writing and to establish an environment that supports physicians in completing their first report. Taken together, this study offers important insights for developing targeted support initiatives to achieve these goals.

## Author Contributions

**Shinsuke Yahata:** writing – review and editing, formal analysis. **Moe Kyotani:** data curation, writing – review and editing. **Hiroyuki Mori:** conceptualization, writing – review and editing, supervision. **Koki Kosami:** writing – review and editing, data curation. **Ryo Fujiwara:** writing – review and editing, data curation. **Ayako Kumabe:** writing – review and editing, data curation. **Naoya Mizutani:** writing – review and editing, data curation. **Tsuneaki Kenzaka:** conceptualization, methodology, writing – original draft.

## Funding

The authors have nothing to report.

## Disclosure

This study was conducted in accordance with the Declaration of Helsinki (as revised in 2013).

## Ethics Statement

This study was approved by the Ethics Committee of the Hyogo Prefectural Tamba Medical Center (approval number: Tan‐I number 1304).

## Consent

All the participants provided informed consent to participate in the study. All participants provided informed consent to participate in the study when they responded online.

## Conflicts of Interest

T.K. is one of the editorial board members of the *Journal of General and Family Medicine*. The authors declare no conflicts of interest.

## Supporting information


**File S1:** Full Japanese version of the questionnaire used to assess perceived barriers to writing case reports, including all survey items and response scales.

**Table S1:** Sensitivity analysis of adjusted odds ratios for barriers to writing case reports using alternative cutoff values.

**Figure S1:** jgf270138‐sup‐0003‐FigureS1‐S16.pdf. **Histogram of cases recognized as suitable for a case report**. Distribution of Likert‐scale scores for the recognition of cases suitable for a case report item stratified by physicians with and without case report writing experience.
**Figure S2: Histogram of having sufficient medical documentation to write a report**. Distribution of responses regarding perceived sufficiency of medical documentation, stratified by case report writing experience.
**Figure S3: Histogram of knowing how to write a case report**. Distribution of Likert‐scale scores for perceived difficulty in knowing how to write a case report, stratified by experience level.
**Figure S4: Histogram of determining the main case points and clinical message**. This figure illustrates the distribution of responses for the item assessing difficulty in identifying the main case points and clinical message, stratified by experience.
**Figure S5: Histogram of lacking a mentor or supporter**. Distribution of perceived lack of mentorship or support, stratified by case report writing experience.
**Figure S6: Histogram of knowing how to search the literature**. Distribution of responses regarding knowledge of how to conduct a literature search, stratified by experience.
**Figure S7: Histogram of knowing how to obtain literature**. Distribution of perceived difficulty in obtaining literature, stratified by experience.
**Figure S8: Histogram of the financial cost of accessing literature**. Distribution of responses regarding perceived financial burden of accessing literature, stratified by experience.
**Figure S9: Histogram of the cost of proofreading**. Distribution of perceived burden related to proofreading costs, stratified by experience.
**Figure S10: Histogram of the cost of publication**. Distribution of responses regarding perceived publication costs, stratified by experience.
**Figure S11: Histogram of having adequate time to write**. Distribution of perceived difficulty in securing adequate time to write, stratified by experience.
**Figure S12: Histogram of lacking motivation to write**. Distribution of responses regarding lack of motivation to write, stratified by experience.
**Figure S13: Histogram of difficulty with English**. Distribution of perceived difficulty with English language proficiency, stratified by experience.
**Figure S14: Histogram of selecting an appropriate journal for submission**. Distribution of responses regarding difficulty in selecting an appropriate journal, stratified by experience.
**Figure S15: Histogram of determining whether ethical review is required**. Distribution of perceived difficulty in determining whether ethical review is required, stratified by experience.
**Figure S16: Histogram of knowing how to apply for ethical review** Distribution of responses regarding difficulty in applying for ethical review, stratified by experience.

**Data S1:** Figure Legends.

## Data Availability

The data that support the findings of this study are available on request from the corresponding author. The data are not publicly available due to privacy or ethical restrictions.
