# Supplementary material for: Barriers and Associated Factors to Writing Case Reports Among Japanese General Medicine Physicians: A Cross‐Sectional Study of the Japan Primary Care Association Members
Source: J Gen Fam Med. 2026 Jun 1;27(4):e70138. doi: 10.1002/jgf2.70138 (PMC13239333; doi:10.1002/jgf2.70138)
Supplement: Supplementary file 1 — File S1: Full Japanese version of the questionnaire used to assess perceived barriers to writing case reports, including all survey items and response scales. [file JGF2-27-e70138-s001.docx]

**Supplementary File 1**

**「ケースレポート論文作成の障壁となる要因」のアンケート**

研究目的：日本プライマリ・ケア連合学会（当学会）学術大会において、他の学術学会と比し、学会発表後の論文化率が極めて低いことが報告されています。当学会員において、臨床研究に比し、比較的論文化しやすいと思われるケースレポート作成について、その障壁となる要因を検討します。結果は今後のわれわれのワークショップ活動、学会発表・研究に役立て、学会員のケースレポート作成に寄与したいと存じますので、宜しくお願い申し上げます。

*必須

アンケートに協力しますか *

- はい

性別 *

- 男性
- 女性
- その他
- 無回答

年齢 *

回答を入力

卒業年数（学生は学年）*

回答を入力

職種（学生は医学科等の学部・学科を記載）*

- 医師
- 看護師
- 薬剤師
- 理学療法士
- 作業療法士
- 言語聴覚士
- 栄養士
- 放射線技師
- 臨床検査技師
- 介護士
- 社会福祉士
- 学生（医学科）
- 学生（保健学科）
- 学生（その他）
- その他：

医師及び看護師の方にお伺いします。主に担当されている診療科は何科ですか。

回答を入力

専門医について、次のうち取得済のものすべてにチェックを入れて下さい。

- 家庭医療専門医
- プライマリ・ケア認定医
- 指導医（プライマリ・ケア連合学会認定）
- 指導医（他の学会認定）
- プライマリ・ケア認定薬剤師
- プライマリ・ケア看護師

主な所属機関の規模 *

- 診療所
- 100床未満の病院
- 100-199床の病院
- 200-399床の病院
- 400床以上の病院
- 大学等の教育機関
- 保健所・行政機関
- その他：

所属機関は初期研修の単独型または管理型臨床研修病院ですか。*

- はい
- いいえ

ケースレポートを書いたことがありますか。*

- はい
- いいえ

書いたことがある方は本数を教えてください。

回答を入力

私どもが行っているワークショップ「ケースレポートを書こう！ Acceptされるために必要なこと」に参加したことがありますか。*

- はい
- いいえ

研究の指導者いますか。*

- はい
- いいえ

文献検索の環境は整っていますか。*

- はい
- いいえ
- どちらでもない

所属施設が研究に関する資金を支出してくれますか。*

- はい
- いいえ
- どちらでもない

外部から研究資金を獲得した経験はありますか。*

- はい
- いいえ

研究支援のSNSグループに所属していますか。*

- はい
- いいえ

実際に経験症例を論文化（ケースレポート作成）するにあたり、障壁と感じていることについての質問です。（0～10段階評価です）＊質問の下に設問の移動ボダンがあります。*

0　　１　　２　　３　　４　　５　　６　　７　　８　　９　　10

　　（0:全く障壁ではない） （５:どちらでもない） 　　（10:とても障害である）

どのような事例がケースレポートに値するかの判断

　　　　〇　　〇　　〇　　〇　　〇　　〇　　〇　　〇　　〇　　〇　　〇

どれくらい診療情報（経過や検査結果等）を集めておけばよいかの判断

　　　　〇　　〇　　〇　　〇　　〇　　〇　　〇　　〇　　〇　　〇　　〇

ケースレポート論文の記載方法

　　　　〇　　〇　　〇　　〇　　〇　　〇　　〇　　〇　　〇　　〇　　〇

論点や臨床メッセージの決定

　　　　〇　　〇　　〇　　〇　　〇　　〇　　〇　　〇　　〇　　〇　　〇

指導者やサポーターの不足・不在

　　　　〇　　〇　　〇　　〇　　〇　　〇　　〇　　〇　　〇　　〇　　〇

文献の検索方法

　　　　〇　　〇　　〇　　〇　　〇　　〇　　〇　　〇　　〇　　〇　　〇

文献の入手手段

　　　　〇　　〇　　〇　　〇　　〇　　〇　　〇　　〇　　〇　　〇　　〇

文献入手にかかる金銭

　　　　〇　　〇　　〇　　〇　　〇　　〇　　〇　　〇　　〇　　〇　　〇

英文校正にかかる金銭

　　　　〇　　〇　　〇　　〇　　〇　　〇　　〇　　〇　　〇　　〇　　〇

論文掲載にかかる金銭

　　　　〇　　〇　　〇　　〇　　〇　　〇　　〇　　〇　　〇　　〇　　〇

論文作成のための時間の確保

　　　　〇　　〇　　〇　　〇　　〇　　〇　　〇　　〇　　〇　　〇　　〇

論文作成のための動機の不足

　　　　〇　　〇　　〇　　〇　　〇　　〇　　〇　　〇　　〇　　〇　　〇

英語への苦手意識

　　　　〇　　〇　　〇　　〇　　〇　　〇　　〇　　〇　　〇　　〇　　〇

投稿先の選択

　　　　〇　　〇　　〇　　〇　　〇　　〇　　〇　　〇　　〇　　〇　　〇

倫理審査の必要性の判断

　　　　〇　　〇　　〇　　〇　　〇　　〇　　〇　　〇　　〇　　〇　　〇

倫理審査の申請方法

　　　　〇　　〇　　〇　　〇　　〇　　〇　　〇　　〇　　〇　　〇　　〇

その他に障壁と感じていることがあれば教えてください。

回答を入力

ケースレポートの作成は重要であると思いますか。*

１ 　２　 ３　 ４　 ５　 ６　 ７　 ８ 　９ 　10

　重要でない ○ 　〇　 〇　 〇　 〇 　〇　 〇　 〇 　〇 　〇　　重要である

ケースレポートの作成に自信がありますか。*

１ 　２　 ３　 ４　 ５　 ６　 ７　 ８ 　９ 　10

　自信がない ○ 　〇　 〇　 〇　 〇 　〇　 〇　 〇 　〇 　〇　　自信がある

その他、「本研究」に関して何かご意見がありましたらお聞かせ下さい。

回答を入力
