# Supplementary material for: Barriers and Associated Factors to Writing Case Reports Among Japanese General Medicine Physicians: A Cross‐Sectional Study of the Japan Primary Care Association Members
Source: J Gen Fam Med. 2026 Jun 1;27(4):e70138. doi: 10.1002/jgf2.70138 (PMC13239333; doi:10.1002/jgf2.70138)
Supplement: Supplementary file 2 — Table S1: Sensitivity analysis of adjusted odds ratios for barriers to writing case reports using alternative cutoff values. [file JGF2-27-e70138-s002.docx]

**Supplementary Table 1**. Sensitivity analysis of adjusted odds ratios for barriers to writing case reports using alternative cutoff values

Logistic regression models adjusted for age and sex

| **Item regarding potential barriers** | **Cutoff score ≥5** | | **Cutoff score ≥6** | | **Cutoff score ≥7** | |
| --- | --- | --- | --- | --- | --- | --- |
|  | **Adjusted OR**  **(95% CI)** | **P-value** | **Adjusted OR**  **(95% CI)** | **P-value** | **Adjusted OR**  **(95% CI)** | **P-value** |
| Recognizing cases suitable for a case report | 5.88 (3.43 to 10.09) | <0.001 | 4.79 (2.91 to 7.89) | <0.001 | 5.22 (3.11 to 8.78) | <0.001 |
| Having sufficient medical documentation to write a report | 9.07 (5.10 to 16.12) | <0.001 | 7.99 (4.73 to 13.50) | <0.001 | 7.31 (4.26 to 12.52) | <0.001 |
| Knowing how to write a case report | 9.37 (5.12 to 17.13) | <0.001 | 6.44 (3.86 to 10.75) | <0.001 | 7.14 (4.22 to 12.10) | <0.001 |
| Determining the main case points and clinical message | 5.25 (2.94 to 9.39) | <0.001 | 3.80 (2.32 to 6.23) | <0.001 | 4.30 (2.65 to 7.04) | <0.001 |
| Lacking a mentor or supporter | 3.02 (1.78 to 5.25) | 0.001 | 1.96 (1.21 to 3.20) | 0.007 | 2.35 (1.46 to 3.79) | 0.001 |
| Knowing how to search the literature | 2.65 (1.64 to 4.29) | <0.001 | 3.13 (1.90 to 5.16) | <0.001 | 3.84 (2.21 to 6.67) | <0.001 |
| Knowing how to obtain literature | 1.72 (1.07 to 2.74) | 0.024 | 2.15 (1.34 to 3.46) | <0.001 | 2.19 (1.34 to 3.59) | 0.002 |
| Financial cost of accessing literature | 1.41 (0.88 to 2.25) | 0.150 | 1.28 (0.80 to 2.05) | 0.301 | 1.20 (0.73 to 1.96) | 0.478 |
| Cost of proofreading | 1.53 (0.94 to 2.47) | 0.087 | 1.19 (0.75 to 1.88) | 0.473 | 1.18 (0.75 to 1.89) | 0.470 |
| Cost of publication | 1.61 (0.99 to 2.760) | 0.052 | 1.04 (0.66 to 1.66) | 0.861 | 0.96 (0.59 to 1.55) | 0.958 |
| Having adequate time to write | 2.15 (1.09 to 4.22) | 0.027 | 2.31 (1.30 to 4.14) | 0.004 | 2.14 (1.28 to 3.57) | 0.004 |
| Lacking motivation to write | 3.66 (2.08 to 6.41) | <0.001 | 3.23 (1.95 to 5.34) | <0.001 | 2.91 (1.80 to 4.71) | <0.001 |
| Difficulty with English | 2.80 (1.64 to 4.78) | <0.001 | 2.24 (1.38 to 3.62) | <0.001 | 2.20 (1.37 to 3.52) | 0.001 |
| Selecting an appropriate journal for submission | 2.72 (1.56 to 4.73) | <0.001 | 2.01 (1.22 to 3.30) | <0.001 | 2.12 (1.32 to 3.42) | 0.002 |
| Determining whether ethical review is required | 2.62 (1.57 to 4.36) | <0.001 | 2.08 (1.30 to 3.34) | <0.001 | 1.92 (1.20 to 3.07) | 0.007 |
| Knowing how to apply for ethical review | 2.60(1.57 to 4.30) | <0.001 | 2.38 (1.48 to 3.84) | <0.001 | 2.20 (1.37 to 3.52) | 0.001 |

Abbreviations: OR, odds ratio; CI, confidence interval.
