# Supplementary material for: Barriers and Associated Factors to Writing Case Reports Among Japanese General Medicine Physicians: A Cross‐Sectional Study of the Japan Primary Care Association Members
Source: J Gen Fam Med. 2026 Jun 1;27(4):e70138. doi: 10.1002/jgf2.70138 (PMC13239333; doi:10.1002/jgf2.70138)
Supplement: Supplementary file 3 — Figure S1: jgf270138‐sup‐0003‐FigureS1‐S16.pdf. Histogram of cases recognized as suitable for a case report. Distribution of Likert‐scale scores for the recognition of cases suitable for a case report item stratified by physicians with and without case report writing experience. Figure S2: Histogram of having sufficient medical documentation to write a report. Distribution of responses regarding perceived sufficiency of medical documentation, stratified by case report writing experience. Figure S3: Histogram of knowing how to write a case report. Distribution of Likert‐scale scores for perceived difficulty in knowing how to write a case report, stratified by experience level. Figure S4: Histogram of determining the main case points and clinical message. This figure illustrates the distribution of responses for the item assessing difficulty in identifying the main case points and clinical message, stratified by experience. Figure S5: Histogram of lacking a mentor or supporter. Distribution of perceived lack of mentorship or support, stratified by case report writing experience. Figure S6: Histogram of knowing how to search the literature. Distribution of responses regarding knowledge of how to conduct a literature search, stratified by experience. Figure S7: Histogram of knowing how to obtain literature. Distribution of perceived difficulty in obtaining literature, stratified by experience. Figure S8: Histogram of the financial cost of accessing literature. Distribution of responses regarding perceived financial burden of accessing literature, stratified by experience. Figure S9: Histogram of the cost of proofreading. Distribution of perceived burden related to proofreading costs, stratified by experience. Figure S10: Histogram of the cost of publication. Distribution of responses regarding perceived publication costs, stratified by experience. Figure S11: Histogram of having adequate time to write. Distribution of perceived difficulty in securing adequate time to wr [file JGF2-27-e70138-s004.pdf]

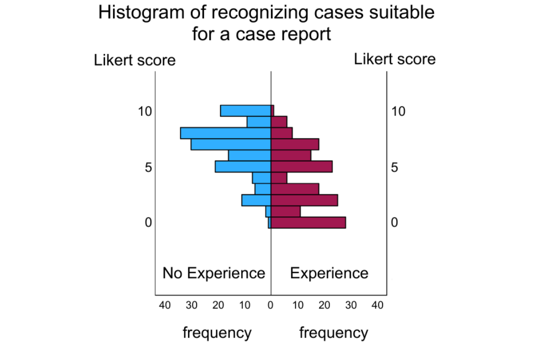

Supplementary_Figure_S1

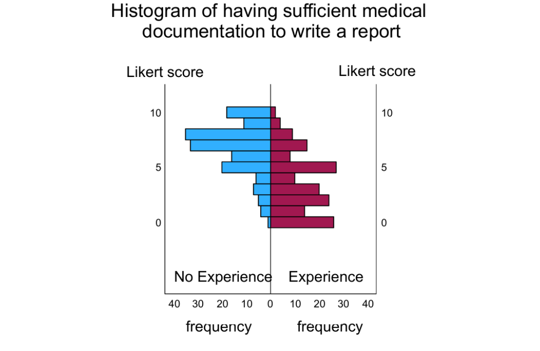

Supplementary_Figure_S2

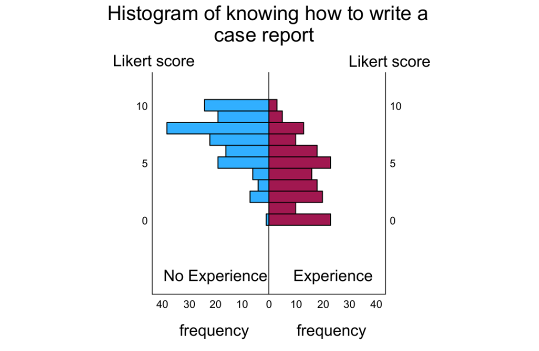

Supplementary_Figure_S3

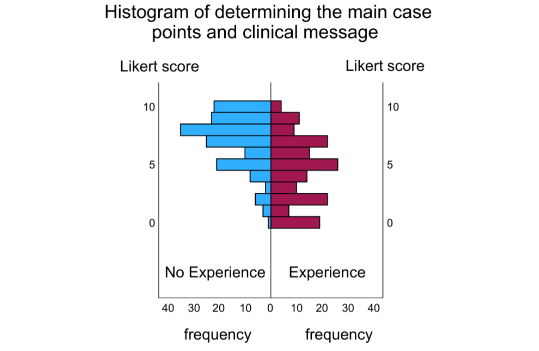

Supplementary_Figure_S4

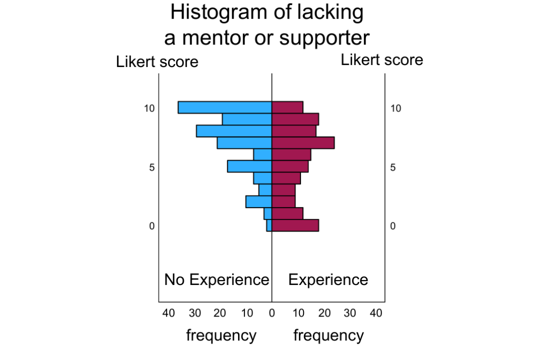

Supplementary_Figure_S5

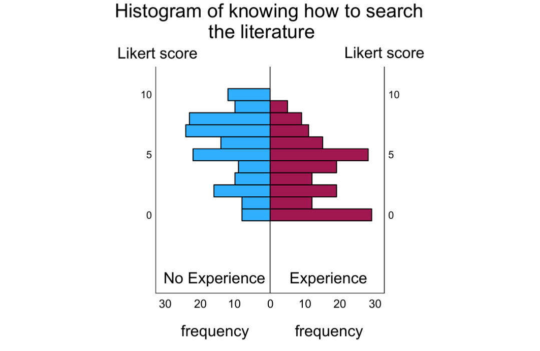

Supplementary_Figure_S6

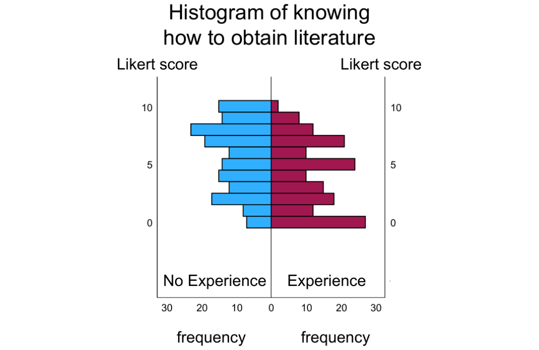

Supplementary_Figure_S7

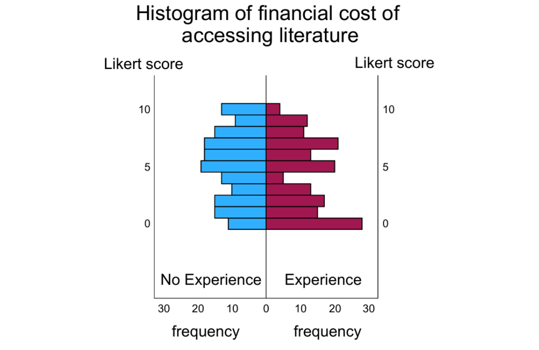

Supplementary_Figure_S8

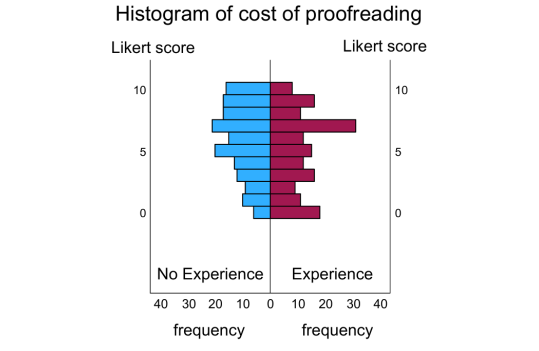

Supplementary_Figure_S9

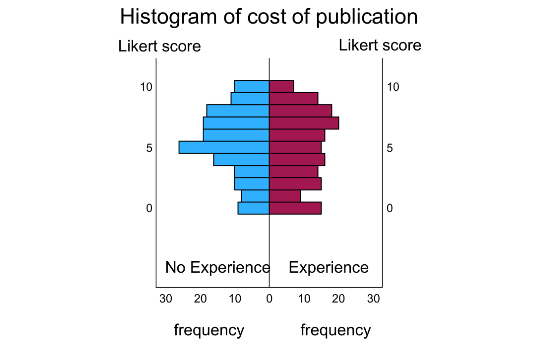

Supplementary_Figure_S10

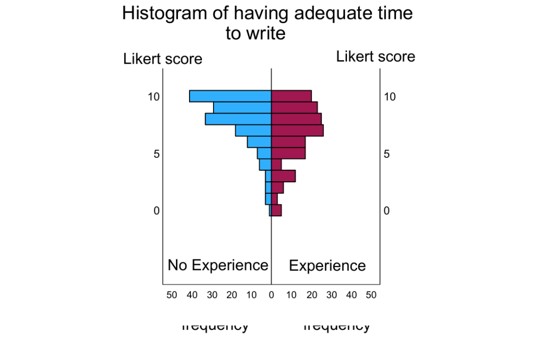

Supplementary_Figure_S11

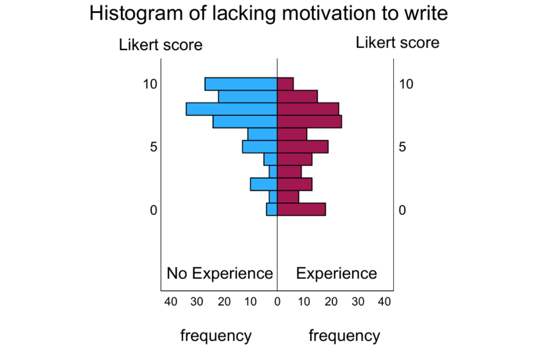

Supplementary_Figure_S12

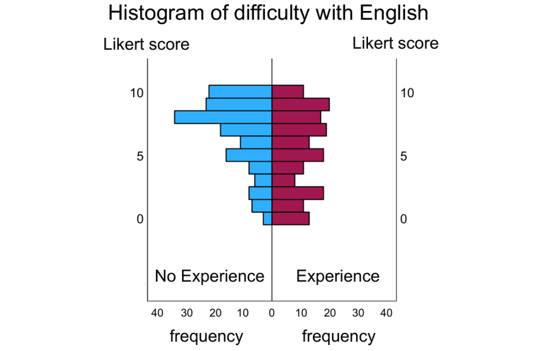

Supplementary_Figure_S13

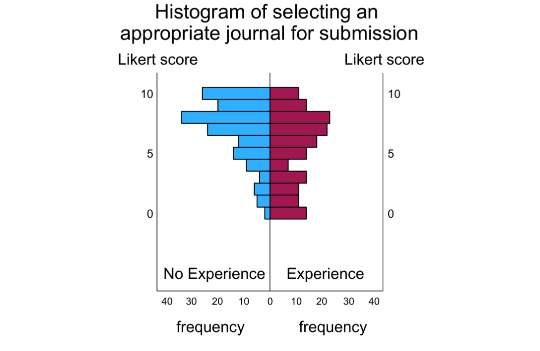

Supplementary_Figure_S14

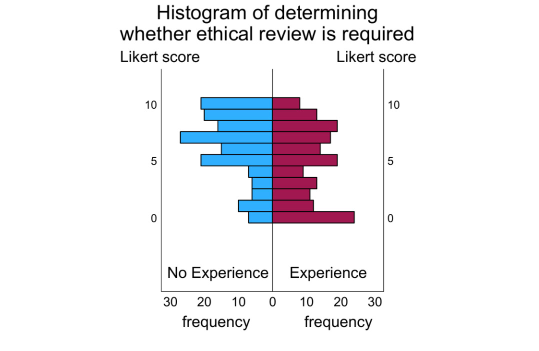

Supplementary_Figure_S15

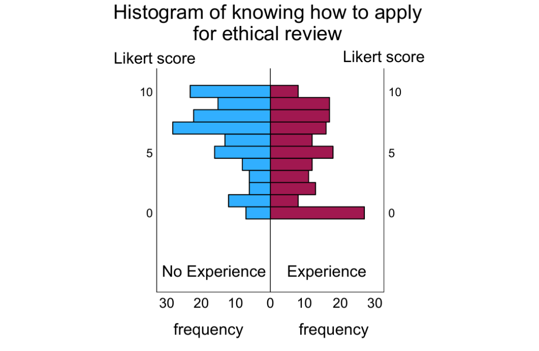

Supplementary_Figure_S16
