# Supplementary material for: Barriers and Associated Factors to Writing Case Reports Among Japanese General Medicine Physicians: A Cross‐Sectional Study of the Japan Primary Care Association Members
Source: J Gen Fam Med. 2026 Jun 1;27(4):e70138. doi: 10.1002/jgf2.70138 (PMC13239333; doi:10.1002/jgf2.70138)
Supplement: Supplementary file 4 — Data S1: Figure Legends. [file JGF2-27-e70138-s003.docx]

**Supplementary Information for**

**Barriers and Associated Factors to Writing Case Reports Among Japanese General Medicine Physicians: A Cross-Sectional Study of the Japan Primary Care Association Members**

**Supplementary Figure S1. Histogram of cases recognized as suitable for a case report.** Distribution of Likert‑scale scores for the recognition of cases suitable for a case report item stratified by physicians with and without case report writing experience.

**Supplementary Figure S2. Histogram of having sufficient medical documentation to write a report.** Distribution of responses regarding perceived sufficiency of medical documentation, stratified by case report writing experience.

**Supplementary Figure S3. Histogram of knowing how to write a case report.** Distribution of Likert‑scale scores for perceived difficulty in knowing how to write a case report, stratified by experience level.

**Supplementary Figure S4. Histogram of determining the main case points and clinical message.** This figure illustrates the distribution of responses for the item assessing difficulty in identifying the main case points and clinical message, stratified by experience.

**Supplementary Figure S5. Histogram of lacking a mentor or supporter.** Distribution of perceived lack of mentorship or support, stratified by case report writing experience.

**Supplementary Figure S6. Histogram of knowing how to search the literature.** Distribution of responses regarding knowledge of how to conduct a literature search, stratified by experience.

**Supplementary Figure S7. Histogram of knowing how to obtain literature.** Distribution of perceived difficulty in obtaining literature, stratified by experience.

**Supplementary Figure S8. Histogram of the financial cost of accessing literature.** Distribution of responses regarding perceived financial burden of accessing literature, stratified by experience.

**Supplementary Figure S9. Histogram of the cost of proofreading.** Distribution of perceived burden related to proofreading costs, stratified by experience.

**Supplementary Figure S10. Histogram of the cost of publication.** Distribution of responses regarding perceived publication costs, stratified by experience.

**Supplementary Figure S11. Histogram of having adequate time to write.** Distribution of perceived difficulty in securing adequate time to write, stratified by experience.

**Supplementary Figure S12. Histogram of lacking motivation to write.** Distribution of responses regarding lack of motivation to write, stratified by experience.

**Supplementary Figure S13. Histogram of difficulty with English.** Distribution of perceived difficulty with English language proficiency, stratified by experience.

**Supplementary Figure S14. Histogram of selecting an appropriate journal for submission.** Distribution of responses regarding difficulty in selecting an appropriate journal, stratified by experience.

**Supplementary Figure S15. Histogram of determining whether ethical review is required.** Distribution of perceived difficulty in determining whether ethical review is required, stratified by experience.

**Supplementary Figure S16. Histogram of knowing how to apply for ethical review.** Distribution of responses regarding difficulty in applying for ethical review, stratified by experience.

**Supplementary File 1.** Full Japanese version of the questionnaire used to assess perceived barriers to writing case reports, including all survey items and response scales.
